# To Prevent Vulcanizers from Leaking Steam

**Published:** 1869-05

**Authors:** J. G. Willis


### TO PREVENT VULCANIZERS FROM LEAKING STEAM.

BY J. G. WILLIS, M. D.

Nearly every person who uses a vulcanizer, in which the thermometer communicates directly with the steam chamber, is more or less annoyed by the escape of steam around the thermometer. This requires the frequent removal of the packing. The cause of this trouble is the great space between the stem of the thermometer and the walls of the tube which contains it, by reason of which at every tightening of the point, by screwing together the parts, the packing is forced into this space, and not enough is retained between the shoulders of the parts to keep the joint steam tight.

Having devised a plan by which this difficulty and annoyance is entirely obviated, I take this method of laying it before the profession, that any who choose may avail themselves of its advantages. In order to be clear in directions, I will designate that portion of the machine which contains the stem of the thermometer, the case, and that portion which contains the bulb of the same, the tube, as it is open at both ends.

First, with a V shaped drill bevel the inner edge of the tube nearly to the outer edge, and in depth about one-eighth of an inch; then file a circular piece of brass to fit the bevel as perfectly as possible; this, when in place, must be on a level with the top of the tube; drill a hole through the center of the brass one-sixteenth of an inch larger than the stem of the thermometer, and it is done.

To apply, invert the case and introduce as much packing as is required, and on the top of the packing place the brass, inverted; now insert the thermometer, and as you place the case over the tube, the beveled piece of brass falls into the countersink in the tube, and furnishes a larger surface for the packing to press upon, and the space of one-thirty-second part of an inch between the stem and brass, although too small for the escape of the packing, is sufficiently large to secure a perfectly tight joint.

I beveled my tube with a common enamel chisel, and completed the whole thing in an hour, and now have the satisfaction of knowing that the escape of steam is impossible, and that portion of my machine will make no further trouble, I think, for years.

